# Influence of Boron Nitride Nanosheets on the Properties of Gelatin–Chitosan Bioinks for Extrusion‐Based 3D Bioprinting

**DOI:** 10.1002/bip.70101

**Published:** 2026-05-08

**Authors:** Aykut Erdogan, Yigitcan Sumbelli, Yapincak Goncu, Nuran Ay

**Affiliations:** ^1^ Graduate School of Education, Eskisehir Technical University Eskisehir Turkiye; ^2^ Chemistry Department Eskisehir Technical University Eskisehir Turkiye; ^3^ Metallurgical and Materials Engineering Department Eskisehir Osmangazi University Eskisehir Turkiye; ^4^ Material Science and Engineering Department Eskisehir Technical University Eskisehir Turkiye

**Keywords:** biofabrication, extrusion bioprinting, hexagonal boron nitride, nanocomposite bioinks, rheology, tissue engineering

## Abstract

In recent years, there has been increasing interest in developing functional bioink systems that better replicate biological and biochemical microenvironments while maintaining high print fidelity and cell viability. This study reports the development and characterization of gelatin–chitosan‐based bioinks reinforced with hexagonal boron nitride nanosheets (BNNSs) for extrusion‐based 3D bioprinting. Structural, chemical, and morphological analyses demonstrated the successful incorporation of BNNSs without the formation of new chemical bonds, while minor shifts in amide bands indicated enhanced hydrogen bonding and physical interactions. Rheological studies revealed that gelatin concentration was the primary factor governing viscosity, whereas BNNSs provided a composition‐dependent reinforcing effect. Notably, at a low shear rate (0.001 s^−1^), viscosity increased from 2865 Pa·s for low concentration gelatin bioinks to 9322 Pa·s for BNNS‐containing high concentration formulations, representing more than a threefold increase. In contrast all formulations exhibited low viscosities below 3 Pa·s at 100 s^−1^, confirming favorable extrusion behavior. Viscoelastic analysis further showed lower tan *δ* values for high gelatin content formulations, indicating elastic‐dominant behavior and improved shape retention at printing temperatures. BNNS incorporation slightly reduced the glass transition temperature by approximately 10°C while preserving blend compatibility, contributing to enhanced thermal responsiveness and more uniform temperature distribution during printing. Printability analysis demonstrated that BNNSs improved shape fidelity in high‐viscosity formulations, yielding printability index (Pr) values close to unity (Pr ≈ 0.94) and stable filament formation. Swelling and degradation studies showed that BNNS‐containing 7.5% gelatin scaffolds exhibited reduced swelling and retained approximately 70% structural integrity after 14 days, whereas low‐polymer formulations underwent rapid degradation. Cell viability assessments confirmed improved fibroblast adhesion and proliferation on BNNS‐containing scaffolds. The incorporation of BNNSs improves the rheological and thermal characteristics of the gelatin–chitosan bioinks and positively influences cell response. These findings suggest that BNNS‐containing formulations provide a more stable and thermally responsive printing platform.

## Graphical abstract

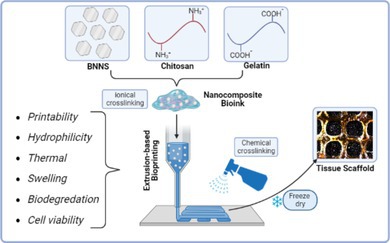

Gelatin–chitosan‐based bioinks reinforced with hexagonal boron nitride nanosheets (BNNSs) are developed for extrusion‐based 3D bioprinting. BNNS incorporation enhances rheological behavior, printability, and thermal response without altering the chemical structure, enabling the fabrication of stable, tissue scaffolds with improved shape fidelity and cell viability.

## 1 Introduction

The global incidence of tissue and organ failure continues to rise due to an aging population and various medical complications. Among clinical interventions, organ transplantation remains the most effective solution for organ failure; however, it relies heavily on donor availability [1, 2, 3]. While some patients can receive suitable tissues or organs in a short period, many must endure years of waiting, significantly decreasing their quality of life and increasing financial burdens [4, 5]. Tragically, some patients lose their lives before receiving compatible organ [1]. Organs or tissues of different species are also used in transplantation as xenografts, but their clinical use is limited due to immune rejection and risks associated with cross‐species transmission of viruses or pathogens [2, 6, 7]. These limitations highlight the urgent need for alternative treatment strategies [8, 9].

Tissue engineering aims to repair damaged tissues or replace them with functional bioengineered constructs [10, 11, 12]. However, replicating the complexity of native tissues, particularly variations in the extracellular matrix (ECM), poses major challenges for fabrication technologies [13, 14]. To address these challenges, multidisciplinary approaches have led to the development of advanced biofabrication methods that aim to mimic native tissue architecture and function [15]. Among these, three‐dimensional (3D) bioprinting has emerged as a powerful platform in tissue engineering and regenerative medicine, enabling the fabrication of complex biological structures with high spatial precision [16, 17]. Current 3D bioprinting technologies include laser‐assisted, inkjet, and extrusion‐based systems. Inkjet bioprinting, which utilizes thermal, mechanical, or piezoelectric forces to deposit droplets, offers high cell viability, rapid printing speed, and cost‐efficiency but is limited to low‐viscosity materials. Laser‐assisted bioprinting is compatible with a broader viscosity range but is costly and requires rapid gelation for precision. In contrast, extrusion‐based bioprinters are well‐suited for depositing highly viscous bioinks and recreating complex tissue structures [18, 19, 20].

The rheological properties of bioinks play a critical role in extrusion‐based printing. Most bioinks exhibit non‐Newtonian, shear‐thinning behavior, where viscosity decreases under applied shear stress. These properties are influenced by polymer concentration and molecular weight, both of which affect shape fidelity and structural integrity after printing [21, 22, 23, 24]. Bioinks may be derived from natural or synthetic biomaterials. In recent years, composite bioinks that integrate multiple components have gained attention due to their improved printability, mechanical strength, and biological properties [8, 25, 26, 27, 28, 29, 30, 31]. Inorganic nanomaterials such as ceramics [32, 33, 34, 35, 36, 37, 38, 39, 40, 41, 42, 43, 44, 45, 46, 47], metals, metal oxides, and metal compounds [48, 49, 50, 51, 52, 53, 54, 55], carbon‐based nanoparticles [56, 57, 58, 59, 60] are increasingly employed to enhance bioink performance [61, 62, 63].

Hexagonal boron nitride (hBN), a structural analog of graphite, has attracted significant interest in biomedical fields due to its unique chemical and physical properties. It has been studied in contexts such as biosensing, tissue engineering, and drug delivery systems [29, 64, 65, 66, 67, 68, 69, 70, 71, 72, 73]. hBN is a layered material with strong in‐plane covalent bonds and weak interlayer van der Waals forces, making it easily exfoliable into boron nitride nanosheets (BNNSs). Owing to its crystalline structure, it is also recognized as an effective solid lubricant [74]. Several studies have shown that hBN‐based additives improve the mechanical and thermal properties of bioinks. For instance, incorporating hBN into PLGA‐ and PLA‐based bioinks enhanced their elasticity and promoted bone tissue regeneration [75, 76]. Functionalized boron nitride nanotubes (f‐BNNTs) have been incorporated into alginate‐based bioinks to enhance their mechanical stability, thermal resistance, and printability in extrusion‐based 3D bioprinting systems [77] Functionalization of BNNSs surfaces enables the tuning of material properties for applications such as drug delivery, mechanical reinforcement, and antibacterial treatments [78, 79, 80].

Chitosan, a naturally derived polysaccharide from crustacean shells, exhibits favorable biological properties including biodegradability, hemostatic effects, antimicrobial activity, and the ability to promote bone formation. However, its limited water retention capacity often causes volumetric shrinkage post‐gelation, requiring it to be blended with other materials [81, 82]. Gelatin, derived from partial hydrolysis of collagen, is biocompatible and biodegradable. It can rapidly form hydrogels and provides cell‐adhesive RGD‐like peptide motifs that facilitate cell proliferation and attachment [83, 84, 85, 86]. Nonetheless, physical or chemical post‐printing crosslinking is necessary to stabilize printed structures. Depending on the method and duration, crosslinking can negatively affect cell viability [87]. The electrostatic interaction between oppositely charged polymers also plays a crucial role in scaffold stability. Chitosan is cationic in acidic environments due to protonated amine groups, while gelatin has amphoteric properties. Their polyelectrolyte complexes contribute to enhanced gelation and mechanical strength, although highly concentrated gelatin–chitosan systems remain challenging to process [88, 89].

Despite various biopolymers being investigated for extrusion‐based bioprinting, maintaining a homogeneous temperature distribution during printing remains a key challenge that directly influences print fidelity, structural integrity, and cell viability. BNNSs, owing to their outstanding thermal conductivity, mechanical reinforcement ability, and inherent biocompatibility, offer a unique opportunity to address this limitation. By facilitating more efficient heat dissipation within the bioink matrix, BNNSs can stabilize the temperature gradients during extrusion, thereby minimizing shear‐induced thermal stress on encapsulated cells and improving the reproducibility of printed constructs.

In this study, BNNSs were incorporated into gelatin–chitosan bioinks to investigate their synergistic effects on rheological behavior, printability, and thermal management during extrusion‐based bioprinting. The resulting BNNS–gelatin–chitosan composite bioinks were further evaluated for their potential to fabricate structurally stable and biologically compatible scaffolds, offering a promising platform for next‐generation tissue engineering applications.

## 2 Materials and Method

### 2.1 Materials

Chitosan (100–300 kDa, > 75% deacetylation) was purchased from ACROS Organics and Bovine gelatin (240 Bloom, Type B) was obtained from Kimbiotek Inc. (Turkiye). hBN powder with average particle size (D50) of 120 nm was supplied by BORTEK Boron Technologies and Mechatronics Inc. (Turkiye). Analytical‐grade chemicals, including acetic acid, sodium hydroxide (NaOH), phosphate‐buffered saline (PBS, 0.01 M, pH 7.4), Calcein‐AM, and propidium iodide (PI), were obtained from Sigma‐Aldrich. The NIH/3T3 mouse fibroblast cell line (ATCC CRL‐1658), derived from NIH/Swiss mouse embryo tissue, was used for in vitro cell viability assessments. All aqueous solutions, including acetic acid, NaOH, and PBS, were sterile filtered using 0.22 μm membrane filters prior to use.

### 2.2 Method

#### 2.2.1 Preparation of BNNSs

hBN was dispersed in distilled water at a concentration of 0.1% (w/v). The suspension was stirred using a magnetic stirrer at 350 rpm for 24 h. The suspension was put in a glass bottle and kept in an ultrasonic water‐bath (35 kHz), at room temperature a period of 5 min. After sonication, the suspension was allowed to settle for 24 h at room temperature. The supernatant was carefully collected in a vessel, while the sediment was dried in an oven at 80°C to determine the concentration of BNNSs in supernatant solution. The dried portion consisting of BNNSs was weighed. The BNNSs concentration in the supernatant solution was found to be 0.076% (w/v).

#### 2.2.2 Preparation of Gelatin–BNNSs Supernatant Solutions

Gelatin was dissolved in BNNSs supernatant solution to prepare gelatin–BNNS mixtures. Gelatin–BNNSs supernatant solution was obtained by adding 2 g of gelatin to 18 mL of BNNSs supernatant and was coded as 5GBNNS. Similarly, gelatin–BNNSs supernatant solution was prepared by adding 3 g of gelatin to 17 mL of BNNSs supernatant and was coded as 7.5GBNNS. During preparation, the mixtures were incubated in a shaking water bath at 40°C and 90 rpm for 1 h to ensure complete dissolution and homogeneous distribution of gelatin. The rheological interactions of gelatin with BNNSs supernatant solution were subsequently evaluated. Gelatin solutions at the same concentrations were prepared in phosphate‐buffered saline (PBS) instead of BNNSs supernatant and were coded as 5G and 7.5G.

#### 2.2.3 Preparation of Chitosan Solution

Chitosan solution was prepared according to the method described by Roehm and Madihally [90]. Briefly, a 5% (w/v) chitosan solution was obtained by dissolving chitosan in 0.25 M acetic acid using a shaking water bath at 60°C and 90 rpm for 6 h. The solution was centrifuged at 4000 rpm for 5 min to remove impurities, and the supernatant was collected and stored at 4°C until further use.

#### 2.2.4 Preparation of Gelatin–Chitosan (GC) and Gelatin–Chitosan–BNNSs (GCBNNS) Bioinks

Gelatin–chitosan (GC) hydrogels were formed at two different concentrations through ionic bonding, as previously described in the literature, to investigate the effect of gelatin concentration [89]. The chitosan solution was mixed in a 1:1 volume ratio with gelatin–PBS solutions (5G and 7.5G) and gelatin–BNNSs supernatant solutions (5GBNNS and 7.5GBNNS). All components were combined at 80°C. The pH of all bioink was adjusted to between 5 and 6 using 1 M NaOH and 10× PBS. The final BNNSs concentration in the chitosan–BNNSs bioinks is 0.038 wt%. A total of four bioink groups were prepared and coded as 5GC and 7.5GC (gelatin–PBS–chitosan), and 5GCBNNS and 7.5GCBNNS (gelatin–BNNSs supernatant solution–chitosan). The resulting nanocomposite bioinks were evaluated in terms of chemical, physical, thermal, rheological behavior, wettability, morphology, biodegradability, and biocompatibility.

#### 2.2.5 Characterization of Components and Nanocomposite Bioinks

X‐ray diffraction (XRD, Rigaku MiniFlex 600) was performed to identify the crystalline phases of hBN. Diffraction patterns were recorded at 40 kV and 15 mA in step mode with a step size of 0.02°, over the 2θ range of 10°–60°. Fourier‐transform infrared spectroscopy (FTIR‐ATR, Bruker Tensor 27) was used to characterize the functional groups of the precursors and nanocomposite bioinks. Measurements were carried out in transmission mode over the wavenumber range of 4000–700 cm^−1^. The thermal behavior of nanocomposite bioinks was examined by differential scanning calorimetry (DSC, Q2000, TA Instruments) over a temperature range of 20°C–300°C, with a heating rate of 10°C/min under a nitrogen flow of 50 mL/min.

#### 2.2.6 Rheological Properties of Nanocomposite Bioink

The rheological behavior of the nanocomposite bioinks, including its viscosity, flow characteristics, and response to mechanical stress, was evaluated using a rotational rheometer (Anton Paar MCR‐102). The bioink samples were placed onto a 5 cm diameter plate, and measurements were conducted using a 1 cm diameter cone with a 1 mm gap at a constant temperature of 25°C. Viscosity was measured over a shear rate range of 0.01–100 s^−1^ to assess shear‐thinning behavior. To determine the viscoelastic properties of the bioink, oscillatory shear tests were performed in amplitude mode with a constant strain rate of 1% and angular frequency of 10 rad/s. The storage modulus (*G*′) and loss modulus (*G*″) were recorded while gradually increasing the temperature from 25°C to 40°C to evaluate the material's viscoelastic response under thermal and mechanical stimuli. Rheological measurements were performed as single runs due to experimental constraints, and the results are presented as representative curves of each formulation.

#### 2.2.7 Production of Scaffolds With 3D Printing and Printability and Characterization

**FIGURE 1:**
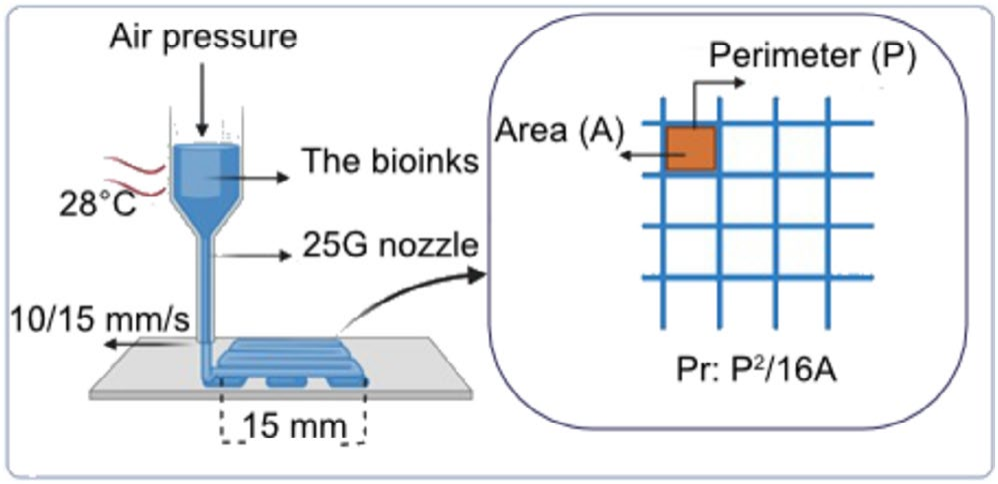
Printing parameters of 3D scaffold and schematic image of bioprinting (designed with BioRender).

Figure 1 shows the printing parameters of the 3D scaffolds and a schematic representation of the bioprinting process. Prior to bioprinting, printing parameters were determined based on a previously optimized protocol using gelatin and chitosan hydrogels [89], employing a 3D extrusion‐based bioprinter (EnvisionTEC 3D‐Bioplotter). Bioinks were tested at a constant temperature of 28°C at pneumatic pressures ranging from 0.5 to 2.4 bar, using either a 25G needle tip or a conical nozzle. Printing speeds of 10, 15, and 20 mm/s were evaluated by extruding straight lines approximately 1 cm in length. The printability of the nanocomposite bioinks was assessed by the width and consistency of the extruded filaments.

Based on printability outcomes, a porous scaffold design (15 mm × 15 mm) with an inter‐fiber distance of 1.5 mm was created using CAD software. Scaffolds were fabricated using different printing speeds and pressure. Printed scaffolds were then imaged using a stereomicroscope (Leica M26), and the printability index (Pr) of the bioinks was evaluated by analyzing the regularity and porosity of the fiber network using the ImageJ software [91]. The printability index was calculated using Equation (1).

(1) $$P_{r}=\frac{P^{2}}{16A}$$

where *P* is the inner perimeter and *A* is the inner area of the pores as seen in the schematic image in Figure 1. After printing, all scaffolds were crosslinked by spraying 1% glutaraldehyde under a fume hood. After 10 min, the scaffolds were washed three times with PBS to remove unreacted glutaraldehyde, as commonly reported in the literature [92, 93, 94]. They were subsequently frozen at −20°C and lyophilized at −70°C under vacuum for 16 h using a freeze dryer (Operon FDB 7003). The scaffolds were stored at 4°C until further characterization.

The thermal behavior of scaffolds was examined by differential scanning calorimetry (DSC, Q2000, TA Instruments) over a temperature range of 20°C–300°C, with a heating rate of 10°C/min under a nitrogen flow of 50 mL/min. Wettability was evaluated by contact angle measurements using a goniometer (Krüss DSA). These measurements were performed on uncrosslinked bioink formulations to assess surface wettability in relation to extrusion behavior and printability. The swelling behavior of the scaffolds was evaluated to assess their ability to absorb and retain biological fluids under physiological‐like conditions. This parameter is critical for determining the structural integrity and porosity retention of the scaffolds, as well as their suitability for nutrient and oxygen diffusion in tissue engineering applications. Dried scaffolds were first weighed (*W*_0_) and then immersed in phosphate‐buffered saline (PBS, pH 7.4) at room temperature for 6 h. This duration was selected to capture the initial and near‐equilibrium swelling behavior of the scaffolds. The swelling test was designed to evaluate the initial water uptake behavior and short‐term swelling capacity of the scaffolds under controlled conditions. It should be noted that these conditions do not fully represent physiological environments. After immersion, samples were carefully removed, surface moisture was gently blotted with filter paper, and the swollen weight (*W*_*t*_) was recorded. The swelling ratio (SR) was calculated using Equation (2).

(2) $$\mathrm{SR}\%=\frac{\left(W_{t}-W_{0}\right)}{W_{0}}*100$$

Biodegradation behavior was assessed by incubating scaffolds in PBS (pH 7.4) at 37°C and 50 rpm for 1, 2, 4, 7, and 14 days. After each incubation, the scaffolds were removed, rinsed gently with deionized water to eliminate residual salts, and dried to a constant weight. The degradation ratio was then calculated based on the change in dry weight using Equation (3):

(3) $$\text{Degredation ratio}\;\left(\%\right)=\frac{W_{0}-W_{t}}{W_{0}}*100$$

where *W*_0_ represents the initial dry weight of the scaffold, and *W*_*t*_ is the dry weight after a given degradation time *t*. The morphology of the scaffolds was examined using a stereomicroscope (Zeiss Stemi 508). For scanning electron microscopy (SEM) analysis, the samples were sputter‐coated with a thin layer of gold–palladium (Au–Pd) using a sputter coater (Agar Sputter Coater) for 20 s and then imaged by SEM (Zeiss Supra 50 VP) at an accelerating voltage of 10 kV.

#### 2.2.8 In Vitro Biocompatibility Testing of Scaffolds

After 3D bioprinting, the cross‐linked scaffolds were lyophilized and assessed for biocompatibility using Live/Dead staining. Prior to staining, the scaffolds were disinfected by incubation with 1% penicillin–streptomycin every other day and washed with phosphate‐buffered saline (PBS). Subsequently, 1.1 × 10^5^ NIH/3T3 mouse fibroblast cells (ATCC CRL‐1658) were seeded onto each scaffold in DMEM F‐12 medium supplemented with 10% fetal bovine serum (FBS) and 1% penicillin–streptomycin. The cell‐seeded scaffolds were incubated at 37°C in a humidified atmosphere with 5% CO_2_ for 7 days. To assess cell viability, a Live/Dead Fixable Dead Cell Staining Kit (ATT Bioquest) was used, with Calcein‐AM (2 μM) for live cell staining and propidium iodide (PI, 4 μM) for dead cells. After staining, the scaffolds were washed with PBS and examined under a fluorescence microscope (Carl Zeiss Axio BioObserver D1) to visualize the stained cells [95, 96].

#### 2.2.9 Statistical Analysis

All data are presented as mean ± standard deviation (SD). Each experiment was performed in duplicate. The mean values were considered for analysis. Statistical significance between groups was accepted at *p* < 0.05.

## 3 Results and Discussion

**FIGURE 2:**
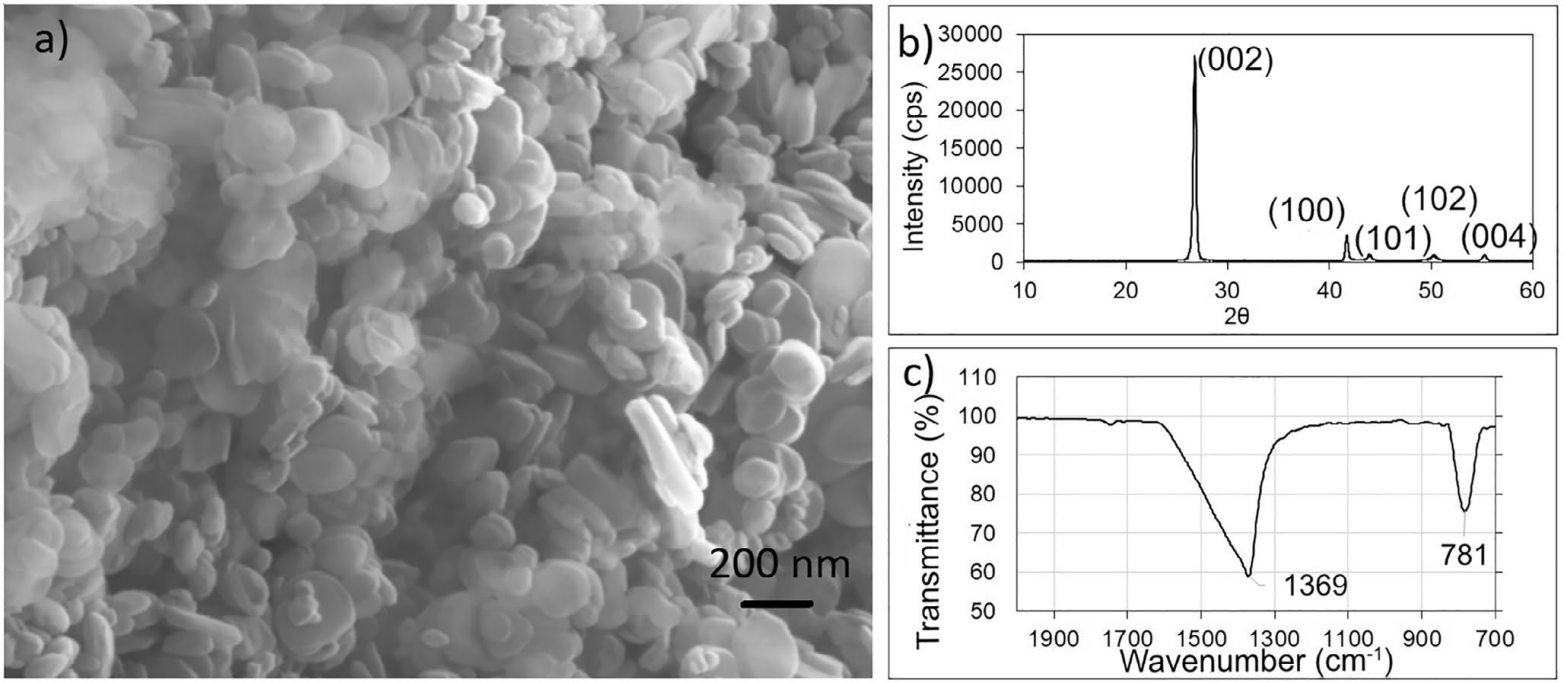
Characterization of the synthesized hBN: (a) SEM micrograph showing the platelet‐like morphology, (b) XRD pattern confirming the hexagonal crystal structure, and (c) FTIR spectrum indicating B—N bonding.

The structural and chemical properties of boron nitride were characterized prior to their incorporation into nanocomposite bioinks. SEM analysis revealed that the precursor powder had a round, flake‐like morphology with smooth surfaces. The average lateral particle size was determined to be approximately 120 nm, indicating the presence of multilayered nanosheets suitable for biomedical applications (Figure 2a). To investigate the crystal structure of hBN, XRD analysis was performed. The most intense diffraction peak was observed at 2*θ* ≈ 26.8°, corresponding to the (002) plane, which is indicative of the layered structure and high crystallinity of hBN. In addition, low intensity but well‐defined peaks were detected at 2*θ* ≈ 41.6° [97], 43.9° [98], 50.3° [99], and 55.2° (004), consistent with the characteristic reflections of hBN (JCPDS PDF card no. 034‐421). The presence of sharp peaks confirms that the starting powder possesses a highly crystalline structure (Figure 2b). FTIR analysis confirmed the presence of characteristic functional groups of hBN, with the B—N stretching vibration identified at 1369 cm^−1^ and the out‐of‐plane B—N—B bending mode observed at 781 cm^−1^ as seen Figure 2c [100].

**FIGURE 3:**
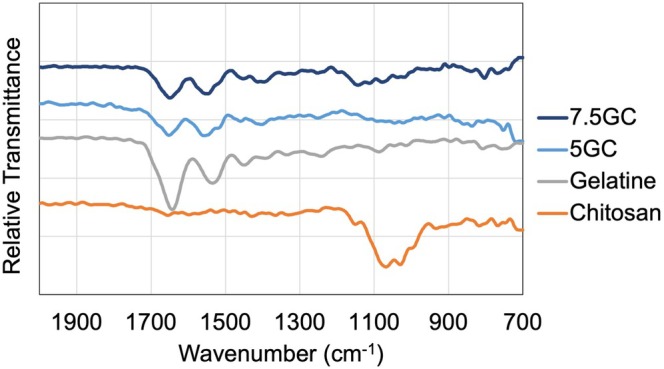
FTIR spectra of gelatin, chitosan, and their corresponding bioinks showing characteristic functional groups in the fingerprint region.

The characteristic functional groups of the components and nanocomposite bioinks were identified by FTIR analysis, and the fingerprint region spectrum is presented in Figure 3. In the gelatin spectrum, the amide II was detected at ~1534 cm^−1^ and the carbonyl (amide I) band at ~1642 cm^−1^ [101, 102]. Pristine chitosan powder, a weak peak was observed at 1150 cm^−1^ and a broad peak at 1065 cm^−1^, corresponding to the C—O—C and C—O vibrations of the polysaccharide backbone. The absence of other well‐defined peaks in the fingerprint region may be attributed to the low sample density, ATR measurement conditions, or residual moisture in the powder [97]. In the GC composite, the amide I band remained essentially unchanged (~1645 cm^−1^), while the amide II band shifted from ~1550 to 1535 cm^−1^, indicating the formation of hydrogen bonds in which —NH groups of gelatins were involved [102].

**FIGURE 4:**
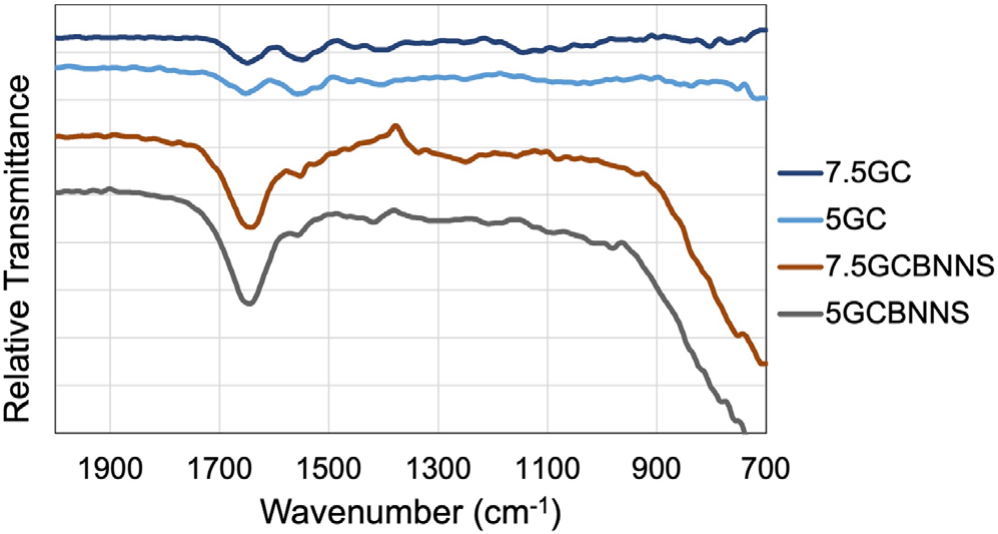
FTIR spectra of GC and GC‐BNNS nanocomposite bioinks showing characteristic functional groups in the fingerprint region.

Comparing the spectrum of 5GC and 7.5GC formulations, an increase in gelatin concentration resulted in slightly enhanced amide I and amide II peak intensities, reflecting a higher content of peptide bonds. Upon the incorporation of BN nanosheets (5GCBNNS and 7.5GCBNNS), no new functional groups indicative of chemical bonding were observed, confirming that BNNSs interact primarily through physical means with the polymeric matrix (Figure 4). Nevertheless, a minor shift and intensity variation in the amide bands suggest enhanced intermolecular interactions and hydrogen bonding between BNNSs and the polymer chains. The characteristic out‐of‐plane B—N—B bending vibration (~780 cm^−1^) is detectable in the BNNSs‐containing samples, reflecting the presence of BN within the matrix. In contrast, the expected B—N stretching vibration (~1369 cm^−1^) is not distinctly visible, likely due to overlap with strong amide III, C—N stretching, and N—H bending modes of gelatin/chitosan, as well as the relatively low BNNSs concentration and possible orientation effects. Such masking of nanoparticle‐derived peaks by polymer absorption has been reported in similar gelatin–chitosan nanocomposite studies. The MWCNT‐reinforced gelatin/chitosan/hydroxyapatite composites where peaks around 1300–1400 cm^−1^ disappeared upon nanoparticle addition [98].

According to the FTIR results, it can be inferred that the interactions between BNNSs and the polymer matrix are primarily physical rather than covalent. The absence of new functional groups indicates no chemical bonding occurs under the current processing conditions. However, minor shifts and intensity changes in amide bands suggest enhanced hydrogen bonding and physical interactions between BNNSs and polymer chains. In combination with electrostatic interactions and hydrogen bonding between gelatin and chitosan, these physical interactions contribute to the formation of a stable composite network. BNNSs act as physical crosslinking points, restricting polymer chain mobility and reinforcing the viscoelastic network, particularly in high gelatin concentration formulations. This synergistic network is consistent with the observed improvements in viscosity, shear‐thinning behavior, and thermal stability. Although porcine gelatin is more commonly used in tissue engineering applications due to its higher bloom strength, bovine gelatin remains a widely accepted alternative. In the present study, the observed trends are primarily governed by polymer interactions and BNNS incorporation, and are therefore expected to be transferable to similar gelatin‐based systems.

### 3.1 Rheological Behavior of Composite Bioinks

**FIGURE 5:**
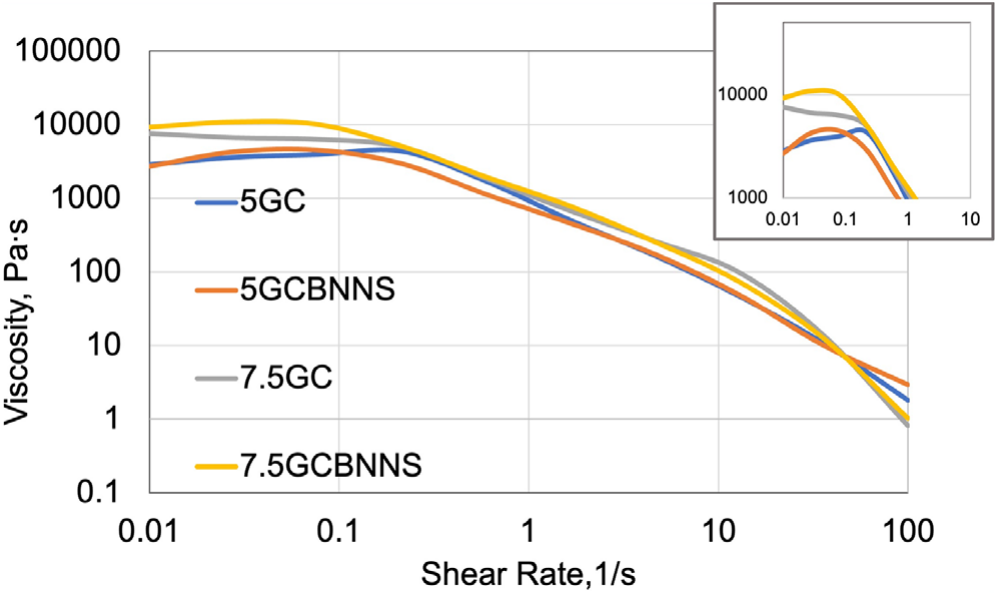
Viscosity of bioink formulations versus shear rate, showing shear‐thinning behavior and effects of gelatin concentration and BNNS incorporation.

The rheological properties of bioinks are key parameters that directly affect the printing process and post‐printing structural stability. The viscosity variation as a function of shear rate at 25°C is presented in Figure 5. The results show that polymer concentration is the dominant factor governing the rheological behavior, while BNNS incorporation provides an additional, composition‐dependent contribution. All bioink formulations exhibit a pronounced shear‐thinning behavior, which facilitates smooth flow during extrusion and ensures sufficient structural integrity for shape retention after printing [99, 103]. Increasing the gelatin concentration resulted in higher viscosity values, likely due to enhanced intermolecular interactions and greater polymer chain entanglement, resulting in the formation of a more robust three‐dimensional network. At a fixed BNNS content, increasing gelatin concentration from 5% to 7.5% increased viscosity at 0.01 s^−1^ from 2865 to 7629 Pa·s, corresponding to *a* ~2.7‐fold increase, while in BNNS‐containing formulations viscosity increased from 2703 to 9322 Pa·s (~3.4‐fold). BNNS incorporation exhibits a concentration‐dependent effect. However, its influence is limited and non‐monotonic at 5% gelatin. In contrast, a pronounced viscosity enhancement is observed at 7.5% gelatin, where BNNS‐containing formulations show higher viscosity values (from 6719 to 10,901 Pa·s), corresponding to an increase of approximately 62%. At high shear rates relevant to extrusion (100 s^−1^), all bioinks displayed low viscosities in the range of 0.8–2.9 Pa·s, confirming favorable flow behavior and the suitability of all formulations for extrusion‐based bioprinting.

**FIGURE 6:**
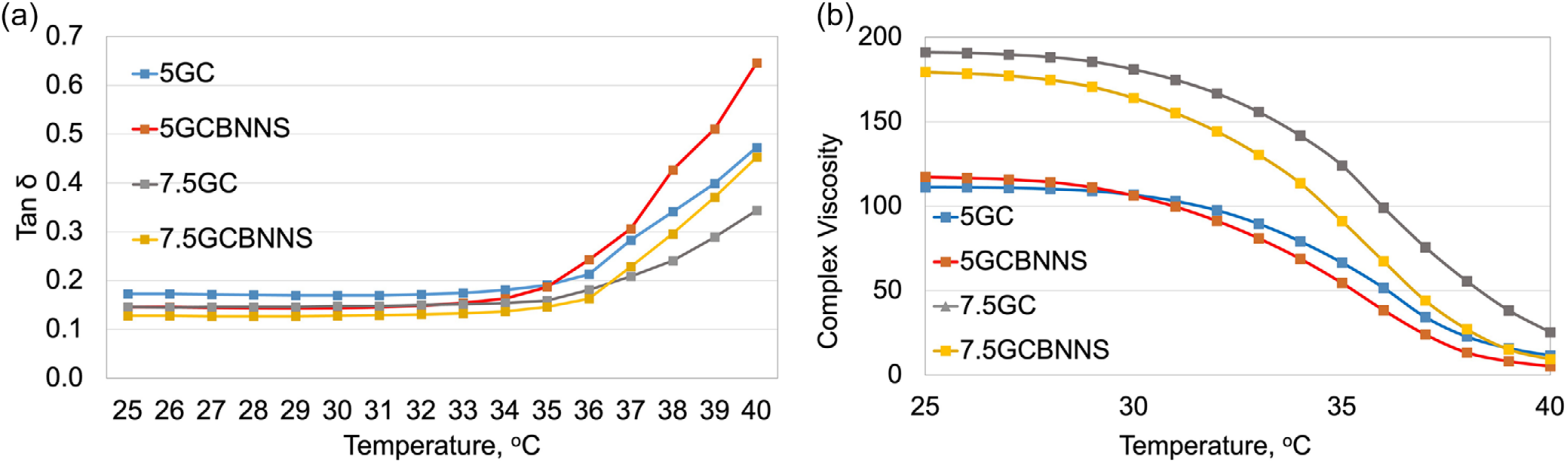
Temperature‐dependent (a) viscoelastic behavior and (b) variation of complex viscosity of the bioink formulations.

The viscoelastic properties of the bioinks were characterized by the *G*′′/*G*′ ratio (Tan *δ*) as seen in Figure 6a. A tan *δ* value lower than unity indicates predominantly elastic (solid‐like) behavior, whereas values higher than unity correspond to more viscous (liquid‐like) behavior. The tan *δ* values of the 7.5GC groups were lower than those of the 5GC samples, indicating that the elastic component was dominant over the viscous component. These results are consistent with previous reports suggesting that higher polymer concentrations enhance shape retention after printing [104].

The effect of BNNSs reinforcement is particularly pronounced at low shear rates and under elevated temperatures. The addition of BNNS contributed to improved pre‐printing stability, particularly at low shear rates. Similarly, the literature reports that layered nanoparticles strengthen the network structure in biopolymer matrices and improve their structural stability [42, 77]. At high shear rates, the convergence of the curves indicates that BNNSs incorporation will not adversely affect the extrusion process. When the temperature‐dependent viscoelastic behaviour was examined, the addition of BNNSs to the 5GC system resulted in a rapid increase in tan *δ* values above 37°C. This suggests that, at low gelatin concentration, BNNS may only partially maintain network integrity, leading the structure to exhibit a more viscous character. In contrast, in the 7.5GC system, BNNS‐containing samples exhibited lower tan *δ* values, which may indicate improved thermal stability. This finding suggests that BNNSs can function as a reinforcing agent at higher polymer concentrations. Fatimi et al. report that maintaining tan *δ* values between 0.2 and 0.6 is critical for achieving an optimal balance between flowability and stability after printing [105]. Low tan *δ* values are advantageous for structural stability during printing. However, as the temperature approaches physiological levels, the increase in tan *δ* suggests a risk of post‐printing hydrogel fluidization.

As shown in Figure 6b, the complex viscosity of the bioink formulations decreases with increasing temperature. This behavior arises from the thermosensitive nature of gelatin–chitosan‐based bioinks. The literature reports that these compositions exhibit a sol–gel transition around 37°C [106, 107, 108]. The higher initial viscosities of the 7.5GC and 7.5GCBNNS groups can be explained by the increased interchain interactions associated with higher polymer concentrations, as also reported in the literature [109]. Furthermore, the 7.5GC BNNS group exhibited higher complex viscosity values compared to 7.5GC, indicating that BNNSs reinforce the physical network structure within the hydrogel matrix, contributing to enhanced structural integrity. In contrast, the 5GC and 5GCBNNS formulations showed lower initial complex viscosities that rapidly decreased with increasing temperature, suggesting that low‐concentration formulations may be less advantageous in terms of post‐printing structural stability. When evaluated together with the tan *δ* results, the pronounced fluidization tendency of the 5GCBNNS group around 37°C highlights the necessity of additional crosslinking strategies for such low‐concentration formulations. Similarly, the literature also reports that supplementary crosslinking approaches, such as pre‐crosslinking, are required for formulations with low polymer concentrations [110]. The 7.5GCBNNS formulation appears to be a promising candidate, as it exhibits relatively low viscosity, low tan *δ* values, and maintains its elastic character with increasing temperature.

The incorporation of boron nitride nanostructures into biopolymer matrices has been reported to enhance rheological properties and print fidelity in extrusion‐based bioprinting. In a related study, boron nitride nanotube reinforced gelatin hydrogel inks exhibited significantly improved viscosity and shear‐thinning behavior compared to pristine gelatin hydrogels, leading to stable filament formation during printing [77]. The enhanced flow behavior was attributed to strong intermolecular interactions between the nanotubes and polymer chains. In the present study, a similar reinforcement effect was observed with boron BNNSs, which promoted structural homogeneity and improved printability, particularly in high‐viscosity gelatin–chitosan systems. It should be noted that rheological measurements were conducted as single runs; therefore, the presented data are interpreted as indicative trends rather than statistically validated differences.

### 3.2 Wettability Behavior of Composite Bioinks

**FIGURE 7:**
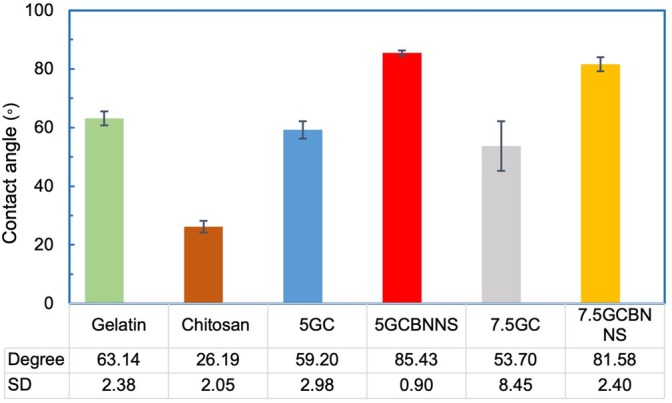
Contact angle degree and droop figures of polymers and nanocomposite bioinks.

Wettability, defined by the hydrophilic or hydrophobic nature of a material's surface, is a critical factor influencing cell adhesion and spreading [99]. According to contact angle measurements (Figure 7), the inherently hydrophobic nature of BNNSs limited the spreading of gelatin and chitosan (both hydrophilic biopolymers) on the material surface, resulting in a more spherical morphology and increased apparent hydrophobicity of the bioink formulations. However, the 7.5GCBNNS, which contained a higher ratio of gelatin compared to the 5GCBNNS, exhibited a reduced contact angle (from 85.43° ± 0.90° to 81.58° ± 2.40°), reflecting enhanced hydrophilicity due to the increased gelatin content. This improved hydrophilicity may contribute to better extrusion behavior and structural integrity during the bioprinting process. Although the BNNSs component contributed to an overall hydrophobic character in the 7.5GCBNNS, changes in surface morphology following bioprinting enhanced its biocompatibility relative to other formulations. These findings are consistent with previous reports indicating that certain structural modifications in hydrophobic materials can significantly improve their biological compatibility [103].

### 3.3 The Results of 3D Bioprinting

The effect of BNNS on the polymer matrix was examined under varying printing parameters, including temperature, pressure, printing speed, and nozzle type. It was observed that at lower printing speeds, filament diameters increased due to transverse spreading, whereas at higher speeds, filaments became thinner and more discontinuous. Therefore, a printing speed of 20 mm/s was found to be unsuitable. The printing speeds of the bioink formulations were optimized according to their increasing viscosities, set at 15 and 10 mm/s, respectively. It was observed that high‐viscosity bioinks required higher extrusion pressures (2.8–3.4 bar) when printed using metal needle‐type nozzles. Therefore, conical plastic nozzles were preferred to reduce the required pressure. The Pr values corresponding to the selected printing parameters for each bioink group are presented in Table 1. The Pr index close to 1 indicates optimal shape fidelity and structural stability after printing [91, 111]. When comparing the bioinks, it was observed that the nanocomposite BNNSs containing formulations could be printed at lower pressures even at high viscosities, suggesting that BNNSs enhance extrusion performance. The addition of BNNSs to the 5GC bioink decreased the Pr value below 1, leading to pore closure and compromised structural integrity. In contrast, incorporating BNNSs into the high‐viscosity 7.5GC bioink improved printing stability, brought the Pr value closer to 1, and reduced the need for excessive extrusion pressure. These results suggest that BNNSs enhance printability and thermal stability even in highly viscous bioinks.

**TABLE 1:** Bioprinting parameters and printability indexes (Pr).

Sample codes	Printing pressure (bar)	Printing speed (mm/s)	Nozzle size and type	Printability index (Pr)
5GC	1.3	15	25G needle type metal	0.997 ± 0.003
5GCBNNS	1	15	25G needle type metal	0.773 ± 0.006
7.5GC	1.8–2.2	10	25G conic type plastic	1.118 ± 0.020
7.5GCBNNS	1.2–1.5	10	25G conic type plastic	0.936 ± 0.095

### 3.4 The Results of Scaffolds

#### 3.4.1 Surface Morphology of Scaffold Structural Assessment of 3D Printed and Nanocomposite Scaffolds

**FIGURE 8:**
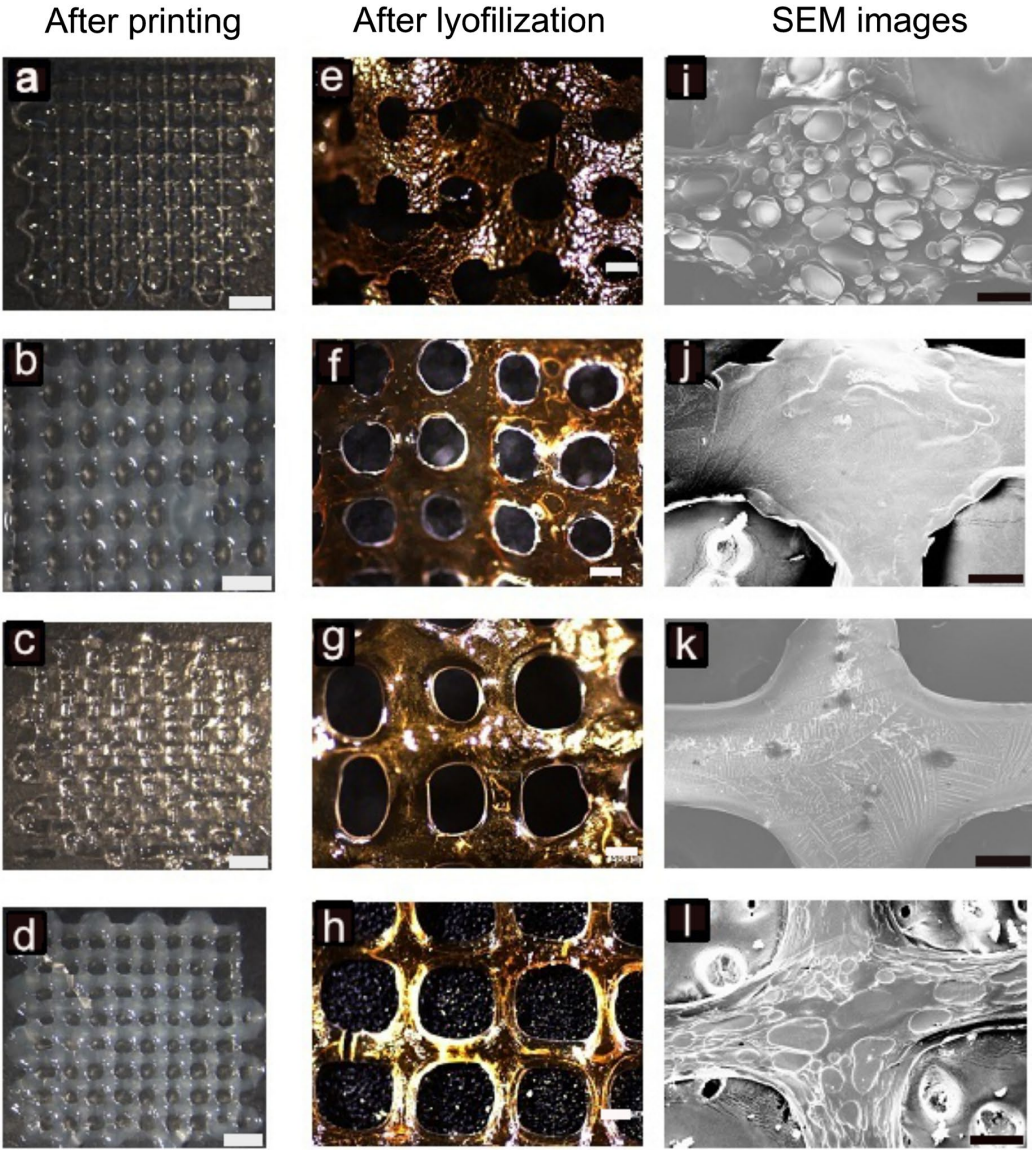
(a–d) Images of printed scaffolds (scale bars: 2 mm); (e–h) stereomicroscopic images of chemically crosslinked and lyophilized scaffolds (scale bars: 500 μm); (i–l) SEM images of crosslinked–lyophilized scaffolds (scale bars: 200 μm). (a, e, i) 5GC, (b, f, j) 5GCBNNS, (c, g, k) 7.5GC, and (d, h, l) 7.5GCBNNS.

Nanocomposite bioinks were printed using the optimized parameters, and the differences in filament stability after printing were compared based on stereomicroscopic and SEM images, as shown in Figure 8. Maintaining printability, defined as the ability of printed filaments to retain their original shape and dimensions without spreading or collapsing, is critical for preserving the integrity of the three‐dimensional architecture [85]. In the 5GC group, the designed square pore geometry was well preserved (Figure 8A), whereas the 5GCBNNS bioink exhibited a noticeable filament expansion after printing (Figure 8B), resulting in the formation of circular pores and partial closure of the structure. Moreover, the filaments in the 5GC group maintained uniform thickness and continuity, indicating minimal spreading behavior. In contrast, the 7.5GC group exhibited poor shape fidelity, showing irregularities, shrinkage, and discontinuities in the printed filaments (Figure 8C). The high viscosity of this formulation likely caused non‐uniform filament diameters, thereby negatively affecting the overall printability. Interestingly, the 7.5GCBNNS group displayed smoother and more stable filaments compared to its BNNSs free counterpart (Figure 8D), and its pore geometry remained nearly square (Pr ≈ 1) as seen Table 1. These observations suggest that the incorporation of BNNS into highly viscous bioinks enhances printability and structural integrity by improving filament formation and reducing post‐print deformation.

During the lyophilization process, the sublimation of ice crystals created pores within the scaffold matrix, facilitating cell adhesion [112]. In the lyophilized images of chemically crosslinked scaffolds (Figure 8), the lower concentration groups (5GC and 5GCBNNS) were found to be more prone to deformation. The initially square pores transformed into circular shapes, and the filament diameters increased under vacuum conditions (Figure 8A,B). In contrast, although the high‐viscosity 7.5GC group exhibited asymmetric pores, the formation of circular pores under vacuum (Figure 8C) without structural fracture was attributed to the stabilizing effect of higher gelatin content and covalent crosslinking. Among all groups, the 7.5GCBNNS scaffold demonstrated the most regular pore geometry after lyophilization (Figure 8D).

SEM images showed that the crosslinked gelatin–chitosan filaments exhibited filament fusion and local thickening at the junction points (Figure 8). The junction regions were considered areas with reduced porosity and increased structural stiffness. No correlation attributable to BNNSs was observed among the samples. In the literature, high porosity in 3D bioprinted hydrogels is regarded as critically important for cell adhesion as well as nutrient and oxygen diffusion [113]. However, it was observed that there were no pores in the connection regions in all samples. This feature suggests that although it increases mechanical stability, it may limit the potential for cellular infiltration and adhesion.

#### 3.4.2 Thermal Behavior of Scaffolds

**FIGURE 9:**
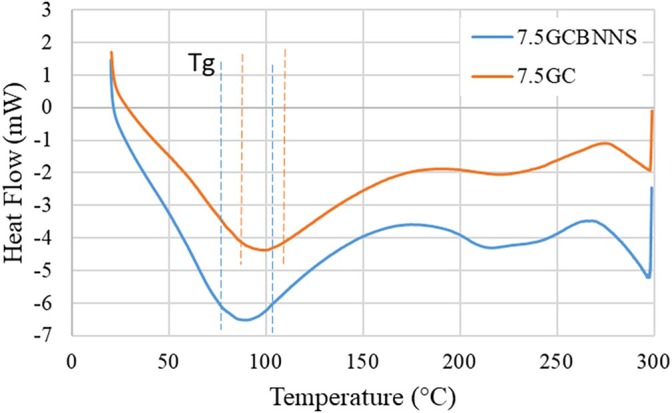
Thermal behavior of bioinks after chemical crosslinking.

The thermal properties of the 7.5GC and 7.5GCBNNS composites were evaluated using differential scanning calorimetry (DSC) (Figure 9). The 7.5GC composite exhibited a single broad glass transition in the 95°C–105°C range. Incorporation of BNNSs led to an approximately 10°C decrease in the glass transition, observed between 85°C and 105°C for the 7.5GCBNNS composite. The presence of a single, broad *T*_g_ in both systems indicates a degree of blend compatibility [114]. The observed decrease in *T*_g_ suggests that BNNSs may slightly increase polymer chain mobility, reducing interchain interactions, which could facilitate a more uniform distribution of heat within the matrix. As a result, thermally induced transitions may occur at lower temperatures while remaining controlled. Maroom et al. reported that the addition of CNTs to nanocomposites, contrary to expectations, led to a decrease in the glass transition temperature (*T*_g_), and they suggested that this behavior is typical of fully exfoliated and homogeneously dispersed nanocomposites [115]. In addition, the increased intensity of the *T*_g_ peak in the BNNSs containing sample indicates a more pronounced thermal response, which may enhance the material's resistance to abrupt temperature changes. This property could be beneficial for maintaining scaffold integrity and supporting cellular activity under processing conditions or physiological temperature fluctuations.

Similar observations have been reported for BNNTs‐reinforced gelatin hydrogels, where the incorporation of BNNTs improved the thermal stability and heat dissipation capability of the bioink during printing and post‐curing processes [77]. The enhanced thermal performance was attributed to the high intrinsic thermal conductivity of boron nitride nanostructures, which facilitated uniform heat transfer and prevented localized overheating within the printed constructs. Consistent with these findings, the presence of BNNSs in the present study contributed to a more homogeneous temperature distribution within the gelatin–chitosan matrix, which is beneficial for maintaining filament shape and minimizing deformation during extrusion‐based bioprinting.

#### 3.4.3 Swelling Behavior of Scaffolds

**FIGURE 10:**
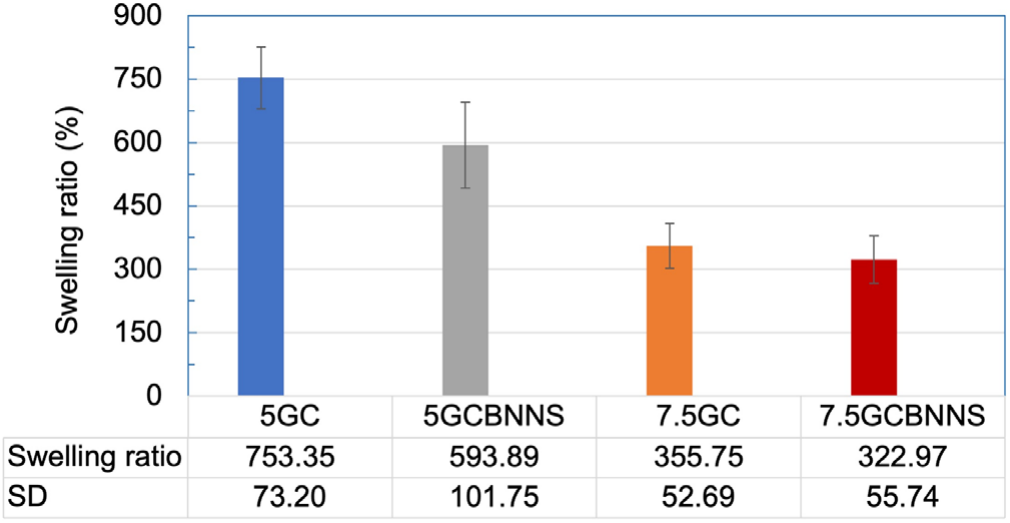
Swelling behavior of the scaffolds in PBS (pH 7.4) at room temperature over 6 h, representing the initial water uptake capacity under controlled conditions.

The swelling test was conducted to evaluate the water absorption capacity of the covalently crosslinked scaffolds. As shown in Figure 10, the 5GC group exhibited a water uptake of 753.35%, while the incorporation of BNNSs reduced this value to 593.89%. The presence of BNNSs decreased the water absorption ability of the scaffolds. When comparing 5GC and 7.5GC, increasing the gelatin concentration resulted in lower water uptake in the crosslinked scaffolds. The 7.5GCBNNS scaffold maintained its internal structural stability and showed resistance to swelling. These results indicate that the scaffolds exhibit composition‐dependent swelling behavior. Formulations with higher water uptake (5GC and 5GCBNNS) show a greater fluid absorption capacity, while higher polymer content (7.5GC and 7.5GCBNNS) results in reduced swelling and improved structural stability. These findings should be interpreted as comparative trends under the tested conditions rather than direct indicators of in vivo or in vitro performance.

#### 3.4.4 Degradation Behavior of Scaffold

**FIGURE 11:**
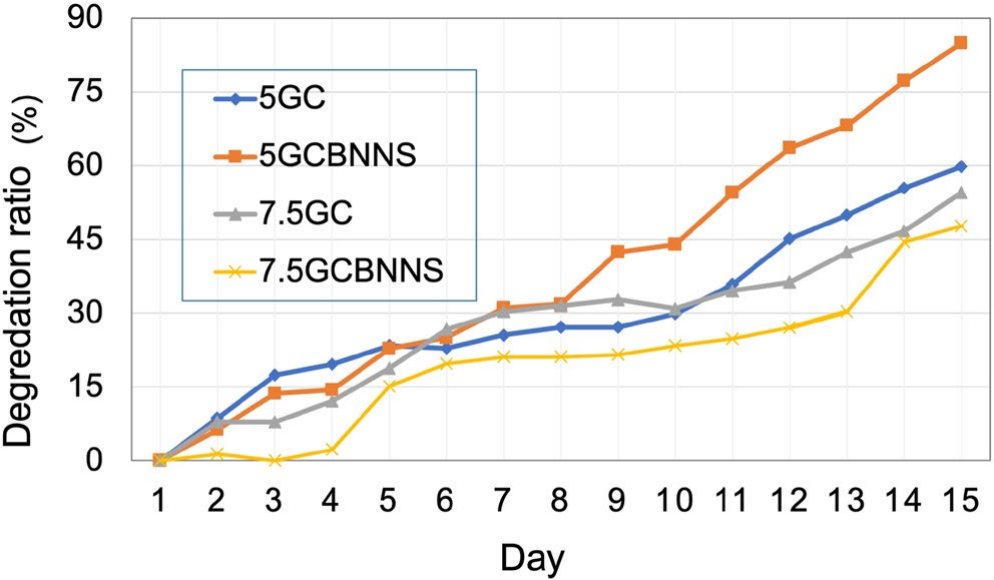
Degradation behavior of the scaffold in PBS (pH = 7.4°C, 37°C).

The degradation profiles of scaffolds were monitored for 14 days under simulated physiological conditions. As illustrated in Figure 11, all groups exhibited approximately 20% mass loss by Day 4. However, long‐term degradation behavior varied significantly. The 7.5GCBNNS group retained the highest structural integrity, with only ~30% degradation by Day 14. In contrast, the 5GCBNNS scaffold showed the most rapid degradation (~85%), potentially due to lower polymer density and increased interstitial spacing induced by BNNSs. These findings suggest that BNNSs enhance long‐term durability in high‐viscosity matrices but may accelerate degradation when incorporated into low‐viscosity systems with insufficient network cohesion.

#### 3.4.5 Cell Viability Assessment

**FIGURE 12:**
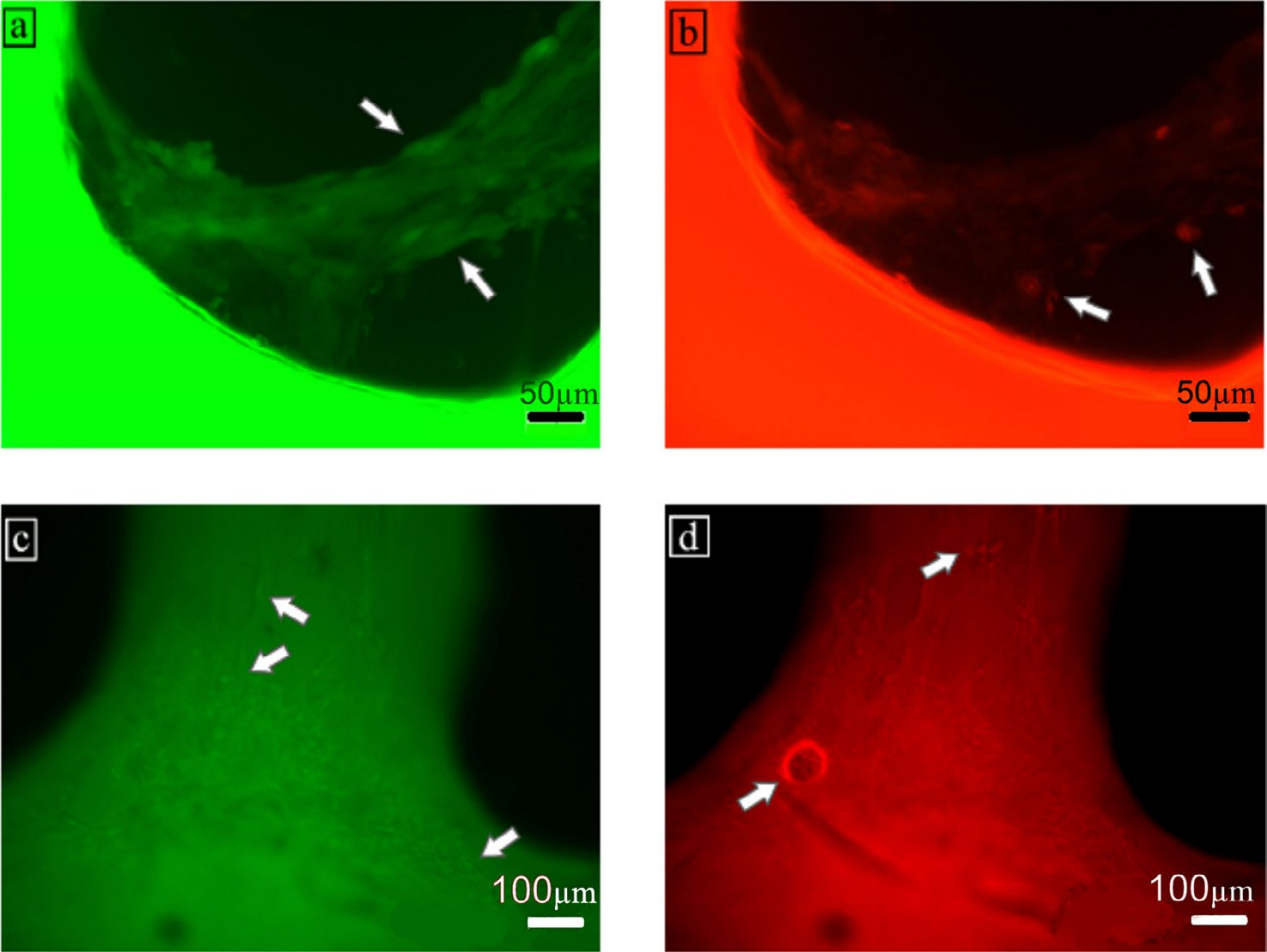
Live (a–c) and dead (b–d) cell staining of scaffolds seeded with 3T3 fibroblasts.

Live/dead fluorescent staining was employed to evaluate the viability of 3T3 fibroblast cells on the scaffolds. As shown in Figure 12, the 7.5GC scaffold (a–b) exhibited cell adhesion and proliferation along the filaments, with some dead cells observed. For the 7.5GCBNNS scaffold (c–d), cells were predominantly localized at filament intersections, with a slightly reduced number of dead cells. These findings indicate that the incorporation of BNNSs did not negatively affect cell viability and may support localized cell proliferation. It should be noted that this evaluation is qualitative, based on Live/Dead staining images. Quantitative assessment of cell proliferation and cytocompatibility using assays such as MTT, WST‐1, or DNA quantification would be required to draw definitive conclusions regarding biocompatibility. Nevertheless, the observed cell adhesion and spreading are consistent with previous reports on BN‐reinforced gelatin or other polymeric scaffolds, which demonstrated enhanced cell‐material interactions attributed to nanostructure‐induced surface roughness and protein adsorption [76, 77, 116, 117]. The BNNS‐containing scaffolds in this study exhibited similar behavior, providing a favorable microenvironment for 3T3 fibroblast attachment and spreading, without evident cytotoxic effects under the tested conditions.

## 4 Conclusion

Gelatin–chitosan‐based bioinks reinforced with BNNSs were successfully developed and evaluated for extrusion‐based 3D bioprinting applications. Rheological analyses demonstrated that polymer concentration is the primary factor governing viscoelastic behavior, while BNNSs provide a composition‐dependent reinforcing effect. At higher gelatin concentration, BNNSs effectively enhanced elastic dominance, thermal stability, and extrusion performance, resulting in improved printability, filament continuity, and post‐printing shape fidelity. Thermal analysis revealed that BNNS incorporation slightly decreased the glass transition temperature while maintaining blend compatibility, indicating enhanced thermal responsiveness and more uniform heat distribution during printing. This thermally adaptive behavior contributed to stable extrusion and minimized deformation under printing conditions. Swelling and degradation studies further confirmed that BNNSs improved long‐term structural stability in high‐viscosity formulations, whereas low‐polymer systems exhibited excessive swelling and rapid degradation. Despite these promising results, the present study has certain limitations. Only a single BNNS concentration was investigated, and the reinforcing effect was found to be strongly dependent on polymer concentration, particularly at lower gelatin contents. In addition, the mechanical performance of the printed scaffolds was inferred from rheological and printability analyses rather than direct compressive testing, and biological evaluation was limited to short‐term in vitro assays. Mechanical characterization could not be performed due to experimental limitations. However, rheological behavior and structural stability provide indirect insight into mechanical performance. Cell viability assessment based on qualitative Live/Dead staining indicated that BNNS‐containing scaffolds supported fibroblast adhesion and spreading, without evident cytotoxic effects under the tested conditions. However, further quantitative assays are required to confirm potential effects on cell proliferation and overall cytocompatibility. BNNSs act as a multifunctional additive that bridges rheological control, thermal regulation, and biological performance when sufficient polymer network integrity is present. These findings position BNNS‐reinforced gelatin–chitosan bioinks as promising candidates for thermally regulated and structurally stable tissue engineering applications.

## Funding

The authors have nothing to report.

## Conflicts of Interest

The authors declare no conflicts of interest.

## Data Availability

The data that support the findings of this study are available on request from the corresponding author. The data are not publicly available due to privacy or ethical restrictions.
